# The use of surfactants in the extraction of active ingredients from natural resources: a comprehensive review

**DOI:** 10.1039/d5ra02072g

**Published:** 2025-07-08

**Authors:** Bhupesh S. Samant, Raja Kaliappan

**Affiliations:** a Nadir Godrej Science Technology and Application Research (NG-STAR) Center, Godrej Chemicals Thane-421506 Maharashtra India bhupesh.samant@godrejinds.com

## Abstract

Surfactants are amphiphilic compounds, crucial in extracting active ingredients from natural resources by enhancing solubility, reducing surface tension, and facilitating phase separation. This review highlights novel extraction techniques, such as micellar extraction, pressurized system extraction, ultrasound-assisted extraction, and microwave-assisted extraction, that leverage surfactants to improve efficiency. It also explores the mechanisms through which surfactants aid in the extraction process, focusing on their application in isolating bioactive compounds from plants, algae, microorganisms, and other natural matrices. We examine the various types of surfactants—anionic, cationic, nonionic, and zwitterionic—used in extraction processes, along with their advantages and limitations. The review also discusses environmentally friendly and sustainable surfactants and assesses the environmental performance of biosurfactants in surfactant-assisted extraction. Finally, we explore potential challenges, including regulatory hurdles, environmental impacts, mass scale-up issues, and the need for further research in the field.

## Abstract 2

This review highlights novel surfactant-based extraction techniques *i.e.* micellar, pressurized, ultrasound, and microwave-assisted, that enhance bioactive compound isolation from plants, algae, microorganisms, and other natural sources.

## Graphical abstract

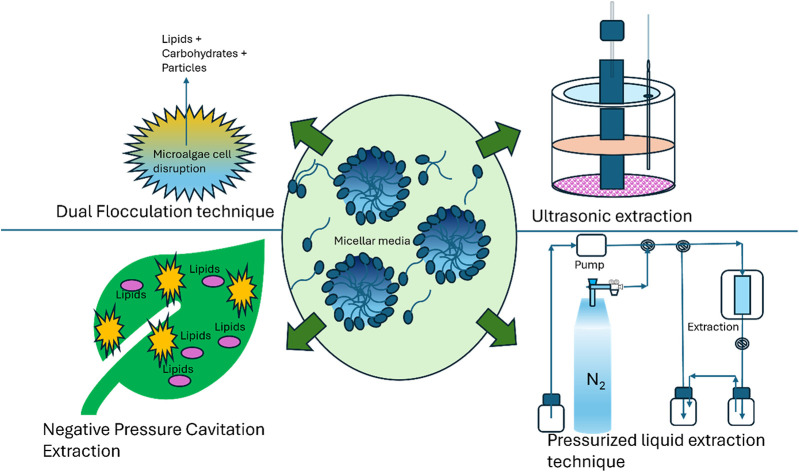

## 1. Introduction

Natural resources, including plants, algae, and microorganisms, contain a wealth of bioactive compounds such as alkaloids, flavonoids, terpenoids, essential oils, and enzymes. These active ingredients are widely used in pharmaceuticals, cosmetics, nutraceuticals, foods/beverages, and agrochemicals.^1^

Efficient extraction of these compounds is crucial for ensuring purity, yield, and bioavailability. Traditional extraction techniques often involve organic solvents, which may pose environmental concerns and may not be as effective in extracting compounds from complex natural matrices. Carbon dioxide (CO_2_) extraction, or supercritical carbon dioxide (SC-CO_2_) extraction, is an advanced method used to extract natural compounds from plant materials and other resources without harsh chemicals. This process is highly selective; however, it has limitations of high cost and the need for expertise to operate the system.^2^

Traditional extraction methods frequently rely on the use of organic solvents such as methanol, ethanol, hexane, or chloroform. While these solvents are often effective in solubilizing a wide range of bioactive compounds, their application raises several concerns, particularly regarding environmental sustainability, human toxicity, and solvent residues in the final product. Moreover, the efficiency of these methods may be limited when dealing with complex natural matrices, such as plant tissues, due to issues such as low selectivity, co-extraction of undesired compounds, and potential degradation of thermolabile constituents during processing.^2^

Deep eutectic solvents (DESs) are a class of green solvents formed by mixing a hydrogen bond donor (HBD) and a hydrogen bond acceptor (HBA) component, typically resulting in a eutectic mixture with a significantly lower melting point than either component.^3^ Due to their tunable physicochemical properties, low volatility, and relatively simple and eco-friendly synthesis, DESs have gained significant attention as alternative solvents for extraction processes in analytical chemistry, environmental remediation, and natural product isolation.^4^ DESs exhibit excellent solvating capabilities for a broad range of compounds, including metal ions, organic pollutants, and bioactive molecules, by altering the molar ratio and type of HBD/HBA used. Furthermore, their non-flammability, biodegradability, and potential for recyclability make them attractive over traditional organic solvents and even some ionic liquids.^5^ However, DESs show limitations of high viscosity, which hinders mass transfer and extraction kinetics. Limited thermal and chemical stability of DESs, which affects their physical properties and extraction performance.^6^ DESs are very sensitive to moisture, this factor creates limitations for natural product extractions. There is incomplete toxicity data for DESs; hence, it creates limitations for the registration of products extracted by using DESs.^7,8^ Because of these limitations, surfactant extraction processes serve as alternative options for natural product extraction.

SC-CO_2_ extraction has emerged as a sophisticated alternative technique for the isolation of valuable phytochemicals and other natural products. In this method, carbon dioxide is brought to its supercritical state, above its critical temperature (31.1 °C) and critical pressure (7.38 MPa), where it exhibits unique properties that combine gas-like diffusivity with liquid-like solvating power. These characteristics make supercritical CO_2_ a highly tunable and selective solvent, capable of extracting specific compounds based on pressure and temperature adjustments.^9^

Unlike conventional organic solvents, CO_2_ is non-toxic, non-flammable, and leaves no harmful residues, thus making the process environmentally benign and suitable for applications in food, pharmaceutical, and cosmetic industries. This method gives high selectivity, minimal thermal degradation, and cleaner extract profiles.^9^ However, SC-CO_2_ extraction systems are capital-intensive, requiring high-pressure equipment and specialized instrumentation. The operation and optimization of these systems demand technical expertise, and the initial cost of setup can be prohibitive for small-scale or resource-limited facilities.

Surfactants, due to their distinctive physicochemical properties, present a promising alternative for enhancing the extraction of bioactive compounds from natural resources. These amphiphilic molecules, characterized by a hydrophilic (water-attracting) head and a hydrophobic (water-repelling) tail, possess the unique ability to reduce surface and interfacial tensions between different phases, such as between solid and liquid phases or between water and organic solvents. This dual affinity allows surfactants to interact with both polar and non-polar substances, making them effective in solubilizing a wide range of bioactive compounds from complex biological matrices.

The presence of surfactants can facilitate the disruption of cell walls and membranes in plant tissues or microorganisms, promoting the release of intracellular contents, including phytochemicals, antioxidants, essential oils, and other natural products. By altering the physicochemical environment, surfactants can enhance mass transfer rates and improve the efficiency of compound extraction, especially when dealing with poorly soluble or hydrophobic molecules.

Additionally, surfactants may be used to form micelles or vesicular structures in aqueous solutions, which can further enhance the solubilization of non-polar bioactive compounds. These micellar aggregates can encapsulate hydrophobic molecules within their core, effectively increasing the bioavailability of the target compounds and facilitating their extraction into the aqueous phase. Moreover, surfactant-mediated extractions are often milder in comparison to traditional solvent-based methods, thus reducing the risk of thermal degradation and preserving the integrity of sensitive bioactive components.

While surfactants offer numerous advantages in terms of enhanced extraction efficiency and selective solubilization, their use also requires careful consideration of factors such as surfactant concentration, type, and environmental impact. High concentrations of surfactants may result in the formation of undesirable by-products or may lead to the saturation of the extraction medium, limiting further extraction. Additionally, the environmental sustainability of surfactants, particularly synthetic surfactants, requires evaluation due to concerns over their biodegradability and potential ecological impact. Nonetheless, the ability of surfactants to effectively solubilize, stabilize, and extract bioactive molecules makes them a valuable tool in the field of natural product extraction, particularly in the context of green chemistry and sustainable processing.

This review focuses on the use of surfactants in such extraction processes, offering insights into their mechanisms, advantages, limitations, and potential applications.

## 2. Properties and types of surfactants

Surfactants, or surface-active agents, consist of a hydrophobic (water-repelling) tail and a hydrophilic (water-attracting) head, which allows them to reduce the surface tension between liquids or between a liquid and a solid. This property makes surfactants particularly useful in the extraction of active compounds from plant or microbial sources.^10^

Based on the charge of the hydrophilic head, surfactants can be classified into four main types.

### 2.1 Anionic surfactants

These surfactants carry a negative charge. Sodium dodecyl sulfate (SDS) is one of the most commonly used anionic surfactants, especially in plant and microbial extraction processes. Anionic surfactants are effective in solubilizing hydrophobic compounds.^11^

Anionic surfactants are characterized by their negatively charged hydrophilic head groups, which confer excellent detergency, foaming, and emulsifying properties. Their structures can be modified to enhance performance, tailor functionalities, or improve environmental compatibility. For example, in hydrophilic head group variations, there were developments done for sulfonates, such as alkylbenzene sulfonates (LAS), where the hydrophilic group is a sulfonate (–SO_3_^−^) attached to an alkyl chain. Linear alkylbenzene sulfonates are preferred over branched ones due to better biodegradability.^12^ Sulfates, *e.g.*, sodium lauryl sulfate (SLS), where the hydrophilic group is a sulfate (–OSO_3_^−^) esterified to an alcohol.^13^ Lastly, carboxylates, such as fatty acid salts, where the hydrophilic group is a carboxylate (–COO^−^) group.^14^

Anionic surfactants are utilized across various industries due to their versatile properties; such as, detergents and cleaners for household and industrial cleaning products; personal care products, such as, shampoos, body washes, and toothpaste for their foaming and cleansing abilities;^15^ emulsion polymerization, as emulsifiers in the production of latex paints and coatings; also, oil recovery processes to reduce surface tension and improve extraction yield.^16^ In pesticide formulations, they enhance spreading and adhesion on plant surfaces.^17^

### 2.2 Cationic surfactants

These surfactants possess a positive charge and are particularly effective in disrupting cellular membranes. Cetyltrimethylammonium bromide (CTAB) is a common example. Cationic surfactants are more selective than anionic surfactants but can also be more toxic, which may limit their use in certain applications.^18^

Cationic surfactants possess a positively charged hydrophilic head group, typically a quaternary ammonium ion (–NR_4_^+^), which imparts their distinctive properties. Their structures can be modified to enhance performance, tailor functionalities, or improve environmental compatibility.^19^

Cationic surfactants are utilized across various industries due to their versatile properties, such as in personal care products, like shampoos, conditioners, and body washes, for their conditioning and antistatic properties. They serve as antimicrobial agents and disinfect and show preservative effects in healthcare and food industries. For targeted drug delivery applications, these can be employed in the formulation of liposomes and nanoparticles. In the textile industry, they are used as softeners and antistatic agents in fabric treatments. They are used in metalworking fluids to prevent corrosion.^20^

For hydrophilic head group variations in cationic surfactants, quaternary ammonium salt is the most common hydrophilic head group, where nitrogen is bonded to four alkyl or aryl groups.^21^ Similarly, pyrrolidinium-based synthesized surfactants showed improved surface activities and applicability in various procedures.^22^

### 2.3 Nonionic surfactants

These surfactants have no charge and are less likely to interact with charged biomolecules, making them useful in delicate extractions where maintaining the structural integrity of compounds is important. Polyethylene glycol (PEG)-based surfactants, such as Tween 20 and Triton X-100, are widely used.^23^

Nonionic surfactants are characterized by their uncharged hydrophilic head groups, which confer excellent emulsifying, wetting, and foaming properties. Their structures can be modified to enhance performance, tailor functionalities, or improve environmental compatibility.^24^ In hydrophilic head group variations, fatty alcohols ethoxylated with ethylene oxide (EO) units, such as alcohol ethoxylates, where the hydrophilic group is a polyoxyethylene chain (–(CH_2_CH_2_O)_*n*_H). The degree of ethoxylation (*n*) influences properties like cloud point and solubility.^19^

Alkyl polyglycosides (APGs), which are derived from glucose and fatty alcohols, these surfactants have a sugar-based hydrophilic head group, offering biodegradability and mildness,^25–27^ for example decyl glucoside. In sorbitan esters, such as sorbitan monooleate, the hydrophilic group is a sorbitan molecule esterified with fatty acids. These are used as emulsifiers in various formulations.^28,29^

Nonionic surfactants are utilized across various industries due to their versatile properties, such as, personal care products, like shampoos, body washes, and facial cleansers, for their mildness and foaming properties.^24^ In household and industrial cleaning, they are employed in detergents and degreasers for their emulsifying and wetting abilities.^30^ In pharmaceutical formulations, they serve as solubilizers and emulsifiers in drug delivery systems.^31^ In the food industry, it is utilized as an emulsifier and stabilizer in products like ice cream and salad dressings. In agricultural formulations, they are incorporated into pesticide formulations to enhance spreading and adhesion on plant surfaces.^32^

### 2.4 Zwitterionic surfactants

These surfactants possess both positive and negative charges, enabling them to maintain a neutral overall charge while interacting with both hydrophobic and hydrophilic compounds. Zwitterionic surfactants like cocamidopropyl betaine are used in gentle extraction processes, particularly in the cosmetic and pharmaceutical industries.^33^

Zwitterionic surfactants typically feature a quaternary ammonium group and a sulfonate, carboxylate, or phosphate group. Their structures can be tailored to enhance performance, stability, and environmental compatibility.

In hydrophilic head group variations, betaine derivatives were prepared by incorporating betaine groups, such as carboxybetaine or sulfobetaine. This improvement enhances water solubility and reduces irritation potential.

Another approach is by mimicking the structure of phospholipids; these surfactants improve biocompatibility and are used in biomedical applications. In polymeric zwitterions, polysulfobetaines, containing quaternary ammonium and sulfonate groups within the same repeat unit, exhibit excellent antifouling properties and are used in ultrafiltration membranes and drug delivery systems due to their biocompatibility and antifouling behavior.

In ionic (anionic, cationic, and zwitterionic) surfactants for hydrophobic tail modifications, chain length alteration in the length of the alkyl chain affects the surfactant's solubility and micelle formation. Incorporating unsaturation or double bonds (*e.g.*, α-olefin sulfonates) can influence the surfactant's biodegradability and foaming properties. Introducing branching of the alkyl chain can impact the surfactant's performance in hard water and its environmental impact.^34^

Incorporation of specific functional groups, such as ethoxylates (ethylene oxide units) to the hydrophobic tail, can enhance water solubility and reduce irritation potential, and also amino acid derivatives like sodium lauroyl sarcosinate offer mildness and biodegradability, making them suitable for personal care products. Incorporating aromatic rings, such as phenyl or naphthyl groups, can enhance hydrophobic interactions and improve performance in high-temperature applications.

Zwitterionic surfactants are utilized across various industries due to their unique properties. Bio-based zwitterionic surfactants derived from fatty acids in non-edible vegetable oils demonstrate excellent interfacial properties, reducing interfacial tension between crude oil and formation water, making them suitable for enhanced oil recovery processes.^35^ Zwitterionic surfactants, such as Super Fat derived from castor oil, are used in emulsion polymerization to produce pressure-sensitive adhesives with improved water resistance and adhesion.^36^ They are also used in shampoos and body washes for their mildness and ability to adjust to different pH levels, offering thickening properties and reducing skin irritation.^37^ These structural modifications enable the tailoring of zwitterionic surfactants to meet specific performance criteria and environmental considerations in diverse applications.

Research on all types of surfactants and their use in the extraction of natural ingredients has been underway for decades; however, their application at the manufacturing scale has yet to be fully optimized. This limited adoption is primarily due to technical constraints such as the robustness of the method, the compatibility of surfactant media with the final extracted product, challenges in sourcing surfactants of consistent quality, especially natural ones, and the reproducibility of results during scale-up studies. These factors collectively impede the widespread industrial use of surfactant-based extraction for natural products. Consequently, various complementary technologies have been explored over the years to enhance the efficiency and reliability of surfactant extraction processes. This review examines these advancements with the aim of increasing awareness and encouraging their adoption across diverse industrial sectors.

## 3. Mechanisms of surfactant-assisted extraction

Surfactants enhance extraction processes through several mechanisms.

### 3.1 Solubilization

Solubilization is a process where surfactants increase the solubility of hydrophobic (non-polar) compounds in aqueous solutions by forming micelles. Micelles are spherical aggregates of surfactant molecules formed when their concentration exceeds the critical micelle concentration (CMC). The hydrophobic tails of the surfactant align inward to form the micelle core, while the hydrophilic heads face the aqueous environment. Hydrophobic compounds are solubilized within the core, allowing them to be dispersed in water. This mechanism is particularly useful for extracting non-polar bioactive substances from plant materials, algae, or microbial cultures where water alone would be ineffective. For example, in the pharmaceutical industry, Tween 80 is used to enhance the aqueous solubility of poorly water-soluble drugs like curcumin or paclitaxel, improving their extraction and bioavailability.^31^ In herbal extractions, solubilization with surfactants helps extract essential oils or terpenes from leaves or flowers.^38,39^

### 3.2 Emulsification

Emulsification involves the formation of a stable emulsion—a dispersed system of two immiscible liquids (typically oil and water). Surfactants reduce the interfacial tension between these liquids and stabilize the dispersion of one phase into another by forming a protective film around the droplets, preventing coalescence. This is particularly advantageous in liquid–liquid extraction processes, where hydrophobic solvents (*e.g.*, hexane or chloroform) are used to extract non-polar compounds from biological matrices. Surfactants ensure effective mixing and interaction between the organic and aqueous phases, improving the mass transfer of target compounds. For example, in the extraction of carotenoids or chlorophyll from plant tissues, emulsifiers like lecithin or Span 20 are used to form oil-in-water emulsions in an aqueous two-phase system for lutein extraction from marigold petals^40^ and carotenoid recovery from tomato wastewater.^41^ Emulsion liquid membrane systems, using surfactants, have been applied for selective extraction of phenolics^42^ and organic acids^43^ from fermentation broths or plant extracts.

### 3.3 Cell membrane disruption

In biological extractions, surfactants disrupt the cell walls or membranes of plants, algae, or microorganisms, facilitating the release of intracellular compounds such as proteins, lipids, and secondary metabolites.

Surfactants interact with the lipid bilayer of cell membranes through hydrophobic and electrostatic interactions. This can lead to permeabilization (increased membrane fluidity) or complete cell lysis, depending on surfactant type and concentration. Disruption of the membrane releases intracellular compounds into the extraction medium. This property is vital for intracellular compound extraction, particularly in the recovery of proteins, lipids, polysaccharides, and secondary metabolites from microbial cells, plant tissues, or algal biomass. For example, SDS is used in the extraction of proteins from *E. coli* by breaking down the bacterial cell wall.^44^ In algal biomass processing, non-ionic surfactants like Triton X-100 aid in lipid extraction for biodiesel production by disrupting the tough cell membranes of microalgae such as *Chlorella vulgaris*.^45^

### 3.4 Reduction of surface and interfacial tension

Surfactants lower the surface tension of liquids and the interfacial tension between immiscible phases (*e.g.*, solid–liquid or liquid–liquid). This enhances wetting, improves solvent penetration, and facilitates mass transfer by increasing the contact area between the solvent and the solid matrix or between immiscible phases. By reducing surface and interfacial tension, surfactants promote better solvent access to plant or microbial structures, allowing more efficient diffusion of target compounds from the matrix into the solvent. For example, in the extraction of polyphenols from green tea or grape skins, the addition of surfactants enhances contact between water–ethanol mixtures and the plant cell walls, leading to faster and more complete extraction. Very recently, in 2025,^46^ the use of Brij S20, a non-ionic surfactant, was investigated to enhance the extraction of polyphenols from grape pomace with optimization study of surfactant concentration, extraction time, pH, and solvent-to-material ratio, resulting in increased total phenolic content and antioxidant activity. A green extraction method utilizing surfactants to recover polyphenols from lotus seedpods was presented.^47^ The approach enhances the extraction process by improving the interaction between the solvent and plant material, leading to higher yields of polyphenolic compounds. In industrial-scale aqueous extractions of herbal actives, surfactants improve wetting of powdered plant material, reducing the extraction time and improving yield.^48^

## 4. Applications of surfactant-assisted extraction

Surfactants have been employed in a wide range of applications for the extraction of active ingredients from natural resources.

### 4.1 Plant extracts

The extraction method and choice of solvent are critical factors in determining the efficiency of target component recovery from plant matrices during the solid–liquid extraction process. Numerous extraction techniques, such as heat-reflux, Soxhlet extraction, ultrasound-assisted extraction, and supercritical fluid extraction (SFE), have been developed for isolating bioactive compounds. However, these methods often require significant time, utilize toxic organic solvents, and are associated with high energy consumption and costs.^49^

**Fig. 1:**
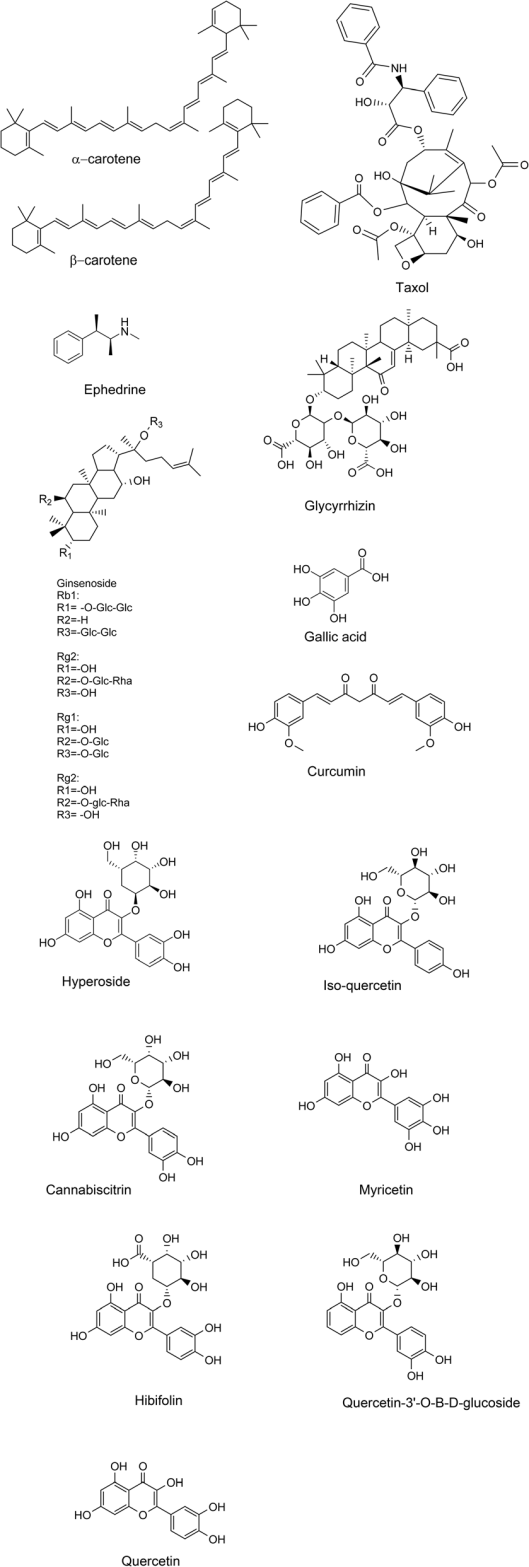
Chemical structures of active ingredients extracted from plant extracts using surfactant media.

Surfactants have been used in the extraction of essential oils, flavonoids, polyphenols, and other bioactive compounds from various plant species. For example, nonionic surfactants like Tween 20 and Triton X-100 are often used to improve the extraction of hydrophobic active ingredients from plant leaves and roots (Fig. 1).^50^

### 4.2 Algal bioactive compounds

Surfactants are essential in extracting lipids, pigments (*e.g.*, carotenoids), and other valuable compounds from algae. The use of surfactants in microalgae extraction can improve lipid yields for biodiesel production and enhance the recovery of pigments for use in cosmetics and nutraceuticals. Algal bioactive extraction is the most explored area for surfactant-assisted extraction technology.^51^

With the growing global need for green biofuels, microalgae have garnered significant attention in the advancement of biotechnology.^52^ The extracellular polymeric substances produced by microalgae are important contributors for their bioproducts, including lipids, carbohydrates, and proteins.^53^ Carbohydrates and microalgal lipids serve as promising resources for efficient biofuel production, such as biomethane, biodiesel, and bio-oil.^54,55^ Additionally, microalgal proteins have potential applications in the pharmaceuticals, home-personal care, and food-beverage industries.^56^ Remarkably, microalgal proteins have been reported to aid in the partial recovery of visual function in blind patients.^57^ However, the production of these high-value bioproducts necessitates the complex major bottleneck processing steps, including harvesting microalgal biomass and disruption of the cell for efficient EP. However, current lipid extraction techniques from wet algae rely on the use of large amounts of organic solvents, especially mixtures of nonpolar and polar solvents.^58^

### 4.3 Marine resources

Extraction of bioactive compounds from marine organisms, such as sponges, corals, and seaweeds, is another growing area where surfactants play a role. Surfactants help to solubilize hydrophobic compounds from these sources, facilitating their use in pharmaceuticals and cosmetics.^59^

### 4.4 Microbial metabolites

Surfactant-assisted extraction is also used for isolating enzymes, antibiotics, and other bioactive secondary metabolites from microbial cultures. Surfactants such as SDS or CTAB can be used to break down microbial cell walls, enabling the recovery of intracellular products.

From various research studies, it has been observed that surfactant micellar extraction exhibits high efficiency, particularly for aromatic and high-molecular-weight natural compounds such as curcumin and Taxol (also known as paclitaxel). To preserve the structural integrity of the extracted natural compounds, researchers have increasingly favored the use of non-ionic surfactants, which mitigate potential deviations in molecular configuration. Moreover, achieving stereochemical selectivity during the extraction process has been identified as a critical factor for ensuring optimal bioactivity in the final application.

Research has demonstrated that several natural compounds, including taxanes such as paclitaxel, baccatin III, and 10-deacetylbaccatin III (10-DAB), undergo degradation at elevated temperatures. Specifically, paclitaxel has been identified as thermally unstable, necessitating its extraction at ambient temperatures. Extraction is commonly performed using a single solvent, such as methanol, or a mixture of methanol and chlorinated methane.^60–62^

A key challenge during room-temperature extraction of these compounds is their limited solubility in polar solvents or pure water. For instance, paclitaxel exhibits poor aqueous solubility.^63^ Though its solubility increases significantly at elevated temperatures. However, this introduces a trade-off between solubility and thermal stability during the extraction process, complicating optimization efforts. To mitigate thermal degradation, employing shorter extraction durations, even at temperatures as high as 150 °C, has been suggested. Alternatively, the incorporation of ionic surfactant micelles presents a promising approach. This strategy enhances solubility in polar solvents or environmentally sustainable aqueous media, enabling efficient extraction at ambient temperatures over extended periods without compromising compound stability.

The role of ionic surfactants and the spatial orientation of surfactant micelles has emerged as a pivotal determinant for extraction selectivity, for example, flavonoids (STF) from *Hibiscus manihot* L. flowers, such as iso-quercetin, hyperoside, cannabiscitrin, myricetin, hibifolin and quercetin-3′-*O*-B-d-glucoside. Studies further indicate that the extraction yield of target natural active ingredients is directly proportional to the concentration of surfactant micelles. However, for effective and scalable mass production, it is essential to optimize the effective surfactant micellar concentration defined as the concentration increment above the CMC. This optimization minimizes undesirable foam generation during large-scale production, which, in turn, enhances yield and reduces operational losses associated with the raw material.

In this context, low-foaming yet highly specific biosurfactants present a promising solution for improving both yield and selectivity of the desired active ingredients. Various surfactant-assisted extraction techniques for natural bioactive compounds from biological sources have been collated in Table 1. While these biosurfactants tend to be more expensive, they offer significant advantages, including enhanced yield and specificity for the desired active compounds, such as ephedrine, glycyrrhizin, and ginsenoside.^64^

**Table 1:** Surfactant-assisted extraction techniques for natural bioactive compounds from various biological sources

Technique	Surfactant used	Methodology	Type of sample	Compound(s) isolated	Ref.
Heat-assisted extraction	—	The conventional method using heat and solvents like methanol or ethanol, limited by thermal degradation of sensitive compounds	Plant material, *Taxus* spp.	Paclitaxel, baccatin III, 10-DAB	49
Soxhlet extraction	—	Continuous extraction using organic solvents under reflux; energy-intensive and not suitable for thermally labile compounds	Dried plant matter	Polyphenols, essential oils	49
Ultrasound-assisted extraction (UAE)	—	Uses ultrasonic waves to enhance cell disruption and improve solvent penetration; can be combined with surfactants	Plant and algal biomass	Flavonoids, lipids, polyphenols	65
Supercritical fluid extraction (SFE)	—	Uses supercritical CO_2_ or co-solvents; eco-friendly but high in cost and complexity	Plant materials, algae, marine sources	Lipids, carotenoids, alkaloids	9
Non-ionic surfactant-assisted extraction	Tween 20, Triton X-100	Nonionic surfactants form micelles to solubilize hydrophobic compounds; suitable for thermolabile bioactives	Plant leaves and roots	Flavonoids, essential oils, polyphenols, curcumin, Taxol (paclitaxel), aromatic polyphenols	24, 51 and 66
Surfactant-assisted lipid extraction	SDS, CTAB	Surfactants disrupt algal cell walls/membranes and solubilize lipids; improves biodiesel precursor recovery	Microalgae (*Chlorella*, *Spirulina*)	Lipids, carotenoids	53
Biosurfactant-assisted extraction	Low-foaming biosurfactants	Eco-friendly, selective solubilization of specific active ingredients; improves compound stability and extraction yield at ambient temperatures	Medicinal plants (*e.g.*, *Ephedra*, *Glycyrrhiza*)	Ephedrine, glycyrrhizin, ginsenoside	64 and 67
Ionic micellar extraction	SDS (anionic), CTAB (cationic)	Ionic surfactants form charged micelles, enhancing aqueous solubility; they also aid in cell disruption and improve selectivity	Microbial cultures, plant extracts	Enzymes, antibiotics, secondary metabolites	68 and 70
Algal EPS extraction	SDS, Triton X-100	Surfactants were used to release EPS (proteins, lipids, carbohydrates) from microalgae, critical for biofuel and biomaterial production	Microalgae	Lipids, carbohydrates, proteins	69 and 70
Surfactant-assisted marine extraction	Tween 80, SDS	Facilitates solubilization of hydrophobic compounds from marine biomass (*e.g.*, sponges, corals); improves recovery of bioactives for cosmetic/pharmaceutical use	Marine organisms (sponges, corals, seaweeds)	Marine alkaloids, lipophilic compounds	68
Surfactant-mediated visual aid extraction	Nonionic surfactants	Extraction of microalgal proteins under mild conditions to retain bioactivity; potential therapeutic use in vision restoration	Microalgae	Bioactive proteins	69
Selective extraction of flavonoids	Ionic surfactants (*e.g.*, SDS)	Surfactants assist in stereoselective extraction from floral tissues; optimization above CMC critical to maximize yield and minimize foaming	*Hibiscus manihot* L. flowers	Iso-quercetin, hyperoside, cannabiscitrin, myricetin, hibifolin, quercetin-3′-*O*-β-d-glucoside	71
Thermal-protected surfactant extraction	Ionic surfactants	Enables extraction at ambient temperature, preserving thermolabile compounds; critical for unstable molecules like taxanes	*Taxus* spp., herbal materials	Paclitaxel, baccatin III, 10-DAB	71

Importantly, the use of biosurfactants aligns with green chemistry principles by promoting environmentally sustainable processes. Additionally, the stability of the extracted natural active ingredients in the presence of biosurfactants during the production process was superior, ensuring the integrity and functionality of the final products.

## 5. Green and sustainable surfactants

**Fig. 2:**
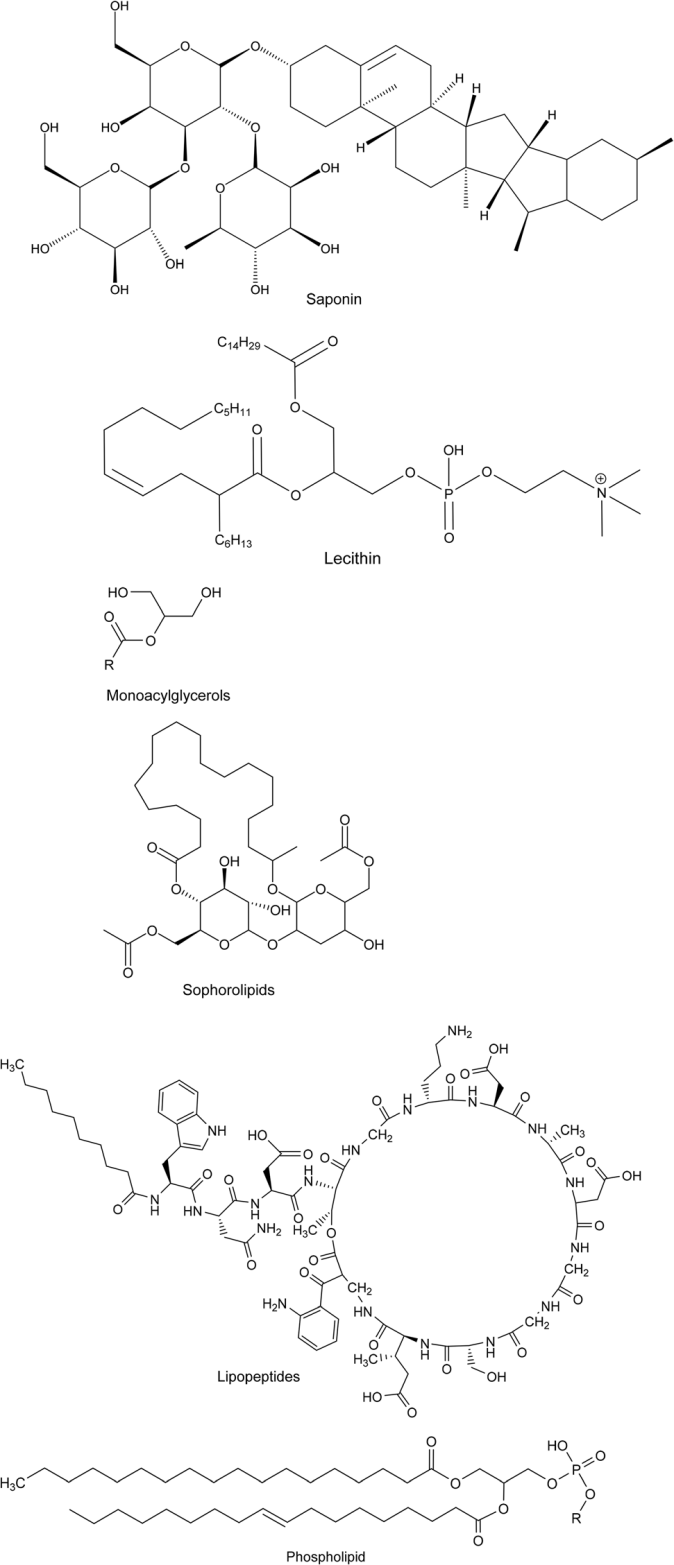
Chemical structures of natural surfactants.

With increasing environmental concerns, the use of environmentally friendly surfactants, or “green surfactants,” has gained significant attention. Biosurfactants, derived from natural sources such as microbial fermentation or plant-based feedstocks, offer a more sustainable alternative to traditional petroleum-based surfactants. Examples include rhamnolipids, sophorolipids, and saponins (Fig. 2). These biosurfactants are biodegradable and exhibit low toxicity, making them ideal for applications where environmental impact is a key consideration.

Ionic liquids, another emerging class of surfactants, are also being explored for their potential in green extraction technologies. Ionic liquids can be tailored to specific applications, and their recyclability makes them a sustainable option in the extraction of bioactive compounds.

In the context of natural product extraction, the pretreatment process preceding the actual extraction is a critical determinant of overall efficiency and product integrity. This stage typically involves drying the raw material under specific temperatures and time conditions. However, improper drying parameters such as excessive durations or elevated temperatures may lead to the thermal degradation of sensitive compounds. Investigating the application of surfactants during this phase offers significant potential. Surfactants could play a dual role by stabilizing natural bioactive constituents during the drying process and enhancing the selective extraction of target compounds from a complex matrix within the pretreated material.

Environmental performance assessment of biosurfactants have gained attention for their environmental advantages in surfactant-assisted extraction processes. These advantages include biodegradability, low toxicity, and renewable sourcing, aligning with the principles of green chemistry. However, traditional environmental metrics, such as the E-factor (environmental factor), atom economy, and carbon footprint, must be applied for an effective sustainability assessment. These metrics offer a means of evaluating the overall environmental impact of biosurfactants in extraction processes, enabling a clearer comparison with conventional surfactants (*e.g.*, synthetic or petrochemical-based surfactants).^72^

In this context, using green chemistry metrics in the evaluation of biosurfactant-assisted extraction can help quantify their sustainability, guide their integration into large-scale operations, and identify opportunities for optimization.^64^

The E-factor is one of the most widely used green chemistry metrics to assess the environmental impact of a chemical process. It is defined as the mass of waste generated per unit of product. In extraction processes, the E-factor helps evaluate the efficiency of material usage, which is crucial for determining sustainability, especially in natural product extractions, where waste can be significant.^73^ The use of biosurfactants can help reduce the volume of toxic organic solvents, which are typically high in mass and contribute significantly to waste generation. In biosurfactant-assisted extraction, solvents are often substituted or reduced, leading to lower waste volumes. For example: in the extraction of bioactive compounds from algae using biosurfactants, if the E-factor is significantly lower than conventional methods (*e.g.*, solvent-based), it indicates a more environmentally friendly process. Biosurfactants may also enable recycling of solvents, further reducing waste.^74^ For example, while setting the potential benchmark, if a conventional surfactant system (*e.g.*, SDS) has an E-factor of 5, a biosurfactant-assisted system may reduce this to 2 or even 1, indicating significant improvements in waste efficiency. The key outcome in terms of biosurfactant-assisted extraction will be the lower E-factor which would directly correlate with improved sustainability.

Atom economy is a metric used to assess how efficiently the atoms in raw materials are incorporated into the final product. The higher the atom economy, the more sustainable the process, as it reduces the need for excessive reagents and generates less waste. The use of biosurfactants, which are derived from natural sources (*e.g.*, plant oils, microorganisms), tends to involve simpler, more atom-efficient processes than synthetic surfactants, which may require petrochemical feedstocks and multiple synthetic steps.^75^ For example, in extracting flavonoids from plant materials, a biosurfactant system may have a higher atom economy compared to an extraction system using petroleum-based surfactants, as it eliminates additional synthesis steps and reduces the overall chemical load. For example, while setting the potential benchmark, biosurfactants derived from renewable biomass can achieve nearly 100% atom economy when they replace synthetic surfactants, which often involve multiple chemical reactions with low atom economy. The key outcome in terms of biosurfactant-assisted extraction will be the would likely increase atom economy, making the process more resource-efficient.^76^

The carbon footprint refers to the total greenhouse gas emissions associated with the production, use, and disposal of a product or process. This metric is particularly important for assessing the sustainability of bio-based processes, such as biosurfactant-assisted extraction. Biosurfactants generally have lower carbon footprints compared to their synthetic counterparts because they are produced from renewable resources and often involve biotechnological processes that are less energy-intensive than petrochemical processes. For example, if a biosurfactant-based extraction system uses microbial fermentation or plant-based raw materials, its carbon footprint could be significantly lower than a synthetic surfactant system, which relies on fossil fuels and chemical synthesis.^77^

The carbon footprint of biosurfactants depends on the source and production method used during the production.^78^ Biosurfactants from microorganisms or plant sources (*e.g.*, rhamnolipids, sophorolipids) produced *via* fermentation processes are low-carbon solutions, compared to synthetic surfactants that require significant energy for synthesis.^72^

The water footprint evaluates the volume of freshwater used throughout a process, which is a critical factor for processes in water-scarce regions. Additionally, biodegradability refers to the ease with which a substance decomposes, minimizing long-term environmental impacts. Biosurfactants are generally more biodegradable and can function in aqueous extraction systems, significantly reducing water use and waste generation compared to traditional solvent-based extractions, which may require large amounts of water for washing and solvent recovery. For example, in the extraction of polyphenols from plant materials using biosurfactants, the process can be optimized to use minimal water, making it particularly suited for water-scarce regions. Additionally, the biodegradability of biosurfactants ensures that no toxic residues accumulate in the environment. The comparison of biosurfactant-assisted extraction with conventional surfactant-based extraction has been collated in Table 2. Synthetic surfactants like SDS can be non-biodegradable, leading to water pollution, whereas biosurfactants are often biodegradable within a few days under natural conditions.^79^

**Table 2:** Comparison metrics for biosurfactant-assisted extraction with conventional surfactant-based extraction

Metric	Biosurfactant-assisted extraction	Conventional surfactant-based extraction
E-factor	Low (efficiency in solvent usage)	High (waste generated)
Atom economy	High (low chemical load)	Low (multiple steps and reagents used)
Carbon footprint	Low (bio-based, renewable resources)	High (petrochemical-based production)
Water footprint	Low (minimal water use)	High (solvent recovery and washing)
Biodegradability	High (fast biodegradation)	Low (slow biodegradation, toxic)

A comprehensive sustainability scorecard can be used to evaluate the overall environmental performance of biosurfactant-assisted extraction. This scorecard can incorporate key metrics such as E-factor, atom economy, carbon footprint, biodegradability, and water footprint.

To further strengthen the sustainability discussion, more life cycle assessments and techno-economic modeling should be conducted to identify potential cost-reduction opportunities and improve the scalability of biosurfactant-based extractions in industrial settings.^80^ While biosurfactants offer numerous environmental advantages, their scale-up requires careful consideration of production costs, biotechnological methods, and waste management. The application of established green chemistry metrics, such as E-factor, atom economy, and carbon footprint, provides a clear framework for assessing the sustainability of surfactant-assisted extraction processes. These metrics help guide decision-making in selecting and optimizing biosurfactants for specific applications, ensuring a balance between economic feasibility and environmental sustainability (Table 2).^72^

## 6. Novel techniques leveraging surfactants

Several innovative extraction techniques that use surfactants have emerged in recent years. These methods often combine surfactants with physical processes to enhance extraction efficiency and reduce processing time.

### 6.1 Micellar extraction

In micellar extraction, surfactants form micelles that encapsulate hydrophobic compounds, facilitating their extraction from aqueous solutions. This method is especially useful for the selective extraction of low-polarity compounds.^50^

The extraction of bioactive compounds from algae has been more extensively studied using surfactant technology than any other class of compounds. Lipid extraction from dry algae is comparatively straightforward; however, drying algal biomass after harvesting requires substantial energy.^81^ Therefore, a promising approach to reduce energy use and costs in algal lipid extraction is directly extracting lipids from wet algae without a prior dewatering step.^82^ Current methods for lipid extraction from wet algae consume large quantities of organic solvents,^83^ particularly mixtures of polar and nonpolar solvents. The comparative summary of advanced extraction techniques for natural products has been collated in Table 3.

**Table 3:** Comparative summary of advanced extraction techniques for natural products

Technique	Extraction yield	Environmental impact	Scalability	Advantages	Limitations	Ref.
Micellar extraction	Moderate to high	Low (aqueous-based, green solvents)	High	• Simple, cost-effective	• Limited to compounds with micelle compatibility	59 and 61
				• Ideal for hydrophobic/thermolabile compounds	• Requires optimization above CMC	
				• Low energy input		
Ultrasound-assisted extraction (UAE)	High	Moderate (water-based, energy use is moderate)	Moderate to high	• Enhanced mass transfer	• Equipment cost	105, 110, 111 and 117
				• Reduced extraction time	• Potential degradation of sensitive compounds due to cavitation	
				• Low solvent usage		
Microwave-assisted extraction (MAE)	Very high	Moderate (fast but consumes energy)	Moderate	• Rapid heating and extraction	• Risk of thermal degradation	112
				• High yield for polar and non-polar compounds	• Equipment cost	
					• Not suitable for all matrices	
Pressurized liquid extraction (PLE)	High	Low to moderate (can use green solvents)	High (at industrial scale)	• Fast and efficient	• Requires specialized equipment	99
				• High throughput	• Not ideal for thermally sensitive compounds	
				• Can automate and scale		
Surfactant microemulsion systems	Moderate to high	Low (biodegradable surfactants, aqueous media)	Moderate	• High solubilizing power	• Complex formulation	113
				• Improved selectivity	• Phase separation issues	
				• Stable system for labile compounds	• Scalability challenges	

The use of these solvents poses significant environmental challenges and increases operational costs.^84^ Consequently, there is a strong incentive to develop solvent-free techniques, or at least methods that minimize solvent use, for lipid extraction from wet algal biomass to make algal biodiesel production safer, more environmentally friendly, and more economically viable compared to conventional fuels.^83^ Although demulsification offers potential as a method to eliminate organic solvents and directly separate algal lipids, studies have shown that algal emulsions form following the degradation of algal cell walls. These emulsions have a complex composition, including neutral lipids, polar lipids, proteins, and other algal constituents, which complicates the extraction of neutral lipids using traditional techniques.^85–87^ Therefore, it is difficult to break algal emulsions and extract the lipids directly.

To reduce the use of organic solvents in wet algal lipid extraction, alternative methods are needed, particularly considering the high toxicity of certain polar solvents that are essential for effective lipid extraction from wet algae. Studies have indicated that surfactants can interact with algal cell membranes and facilitate cell wall disruption.^88^ By breaking down algal membranes, surfactants have shown promise as effective aids in lipid extraction.^51^ Moreover, algal suspensions naturally contain a variety of surfactants, such as monoacylglycerols, phospholipids, sophorolipids, and lecithin, lipopeptides (for chemical structures refer Fig. 2).^89^ Although these natural surfactants may not be directly effective for lipid extraction, their properties can be modified, or additional surfactants can be produced through saponification reactions to assist in lipid extraction. If surfactants, especially those derived from algae, are effective, this could significantly reduce the need for organic solvents in algal lipid extraction (Table 3).

Recently large number of research articles have been published on the use of cationic surfactants (MTAB, DTAB, and CTAB) for microalgal harvesting and cell disruption.^69,70^ The hypothesis is, that cationic surfactants with positive charge increase biomass aggregation and harvesting efficiency through both hydrophobic interaction mechanisms and electrostatic repulsion.^90^ Also, surfactants can release high-value bioproducts such as lipids, carbohydrates, and proteins from EPS into aqueous solutions through cell disruption.^69,91^ It was noted in these studies that the surfactant concentration above CMC is necessary to achieve efficient biomass harvesting and cell disruption. Also, low efficiency was observed due to the slow rate of biomass flocculation even at high dosages of surfactants.

**Fig. 3:**
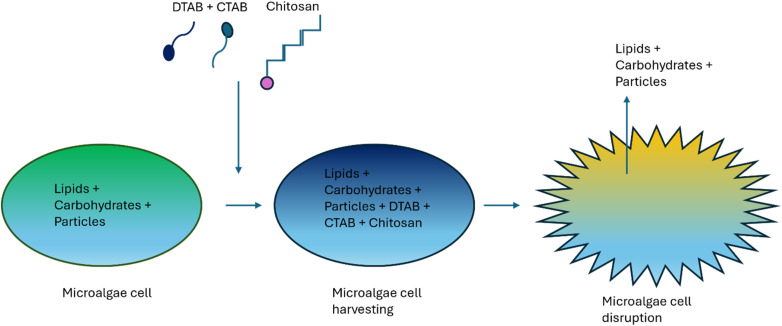
Representation of the dual flocculation technique.

Some research showed improved flocculation efficiency with a low required surfactant concentration for microalgal harvesting and cell disruption^90^ by adjusting surfactant concentration and pH.^92^ It was observed that pH is directly proportional to the harvesting efficiency of CTAB. At pH from 8 to 12, harvesting efficiency was observed to increase from 88% to 98% for CTAB surfactant concentration 50 mg L^−1^ from 400 mg L^−1^. However, high alkalinity (pH 12) created issues for the final application of the biomass and the recycling of culture media for downstream processing.^69^ A research article published in 2018 by Zhong *et al.*^90^ presented the synthesis process of a new surfactant, TPE–DTAB, with ultralow CMC to improve *Chlorella vulgaris* flocculation. To overcome these drawbacks, a novel dual flocculation approach combining chitosan flocculant with cationic surfactants for efficient biomass harvesting and cell disruption, respectively, was recently presented (Fig. 3).^92^

While various techniques have been developed to extract lipids from algae, only a few studies have explored using surfactants, particularly algal-based surfactants, as substitutes for organic solvents to aid in lipid extraction. This is because of the lack of availability of consistent quality algal surfactants and the difficulty in optimization and scaling-up studies (Table 3).

The extraction efficiency was influenced by reaction conditions such as reaction time, pH, and temperature. Surfactants successfully served as substitutes for polar organic solvents in the extraction process of algal lipids. The study showed that while the yield with hexane and ethanol reached only 60.5%, using hexane with algal-based surfactants and hexane with oligomeric surfactants yielded the highest extraction of algal lipids at 78.8% and 78.2%, respectively. Furthermore, the saponifiable lipids extracted with algal-based surfactants and hexane, or with oligomeric surfactants and hexane, represented 78.6% and 75.4% of total algal lipids, respectively. A 10% increase in efficiency was observed over hexane and ethanol extraction.^51,93^

Micellar extraction technology has been studied to extract active compounds from plants for many years. In 2017, a solid–liquid extractions study was carried out with stirring and gentle heating (*T* ≤ 30 °C) conditions, using micellar polysorbate 80 (a non-ionic surfactant) within the solvent system for extracting curcumin from turmeric (*Curcuma longa* L.). The independent variables tested included ethanol concentration (% v/v), extraction duration (min), and surfactant concentration in the solvent (% w/v). Optimal extraction conditions were identified at 59 minutes, with 29% v/v ethanol and 5% w/v surfactant. The curcumin concentration in the extracts was influenced by both the type and concentration of the surfactant (% w/v). Solvents containing the surfactant produced higher curcuminoid levels compared to those with ethanol alone.

Chlorophylls and carotenoids are biosynthesized during photosynthesis and are classified as organic intracellular pigments within the tetraterpene family, comprising eight isoprene units, and are located in chloroplasts and chromoplasts.^94^ A key challenge lies in developing efficient, cost-effective extraction and purification methods, ideally from fresh biomass that preserves the bioactivity and structural integrity of these compounds.^59^ Currently, the predominant approach for carotenoid extraction employs conventional solvents (primarily ethanol)^95^ to solubilize carotenoids from solid biomass, followed by chromatographic techniques,^96^ to isolate the fucoxanthin fraction. However, many existing systems fall short in achieving the desired purity of the final product, which is typically dictated by its intended application. Furthermore, these methods are often complex, demanding significant time and energy inputs, and involve harsh processing conditions.^97^

In 2018, aqueous solutions of different surface-active ionic liquids and anionic surfactants were evaluated for the carotenoid extraction.^59,61^ The lytic effects of two commonly used surfactant families on the cell walls of the microalga *Tetraselmis suecica* were investigated. Aqueous solutions of SDS were selected as the media for optimization. They proposed an integrated process leveraging non-ionic surfactants both as organic solvents and, most importantly, cell-disrupting agents to extract intracellular antioxidants such as carotenoids. Antioxidant extraction was explored using aqueous two-phase systems, with various salting-out agents, including inorganic and organic sodium salts, in aqueous solutions containing the selected surfactants. Extraction efficiency was evaluated for key biomolecules previously identified in this microalga: gallic acid, β-carotene, and α-tocopherol.^98^

Extraction parameters like, concentration, solid–liquid ratio, and time of extraction were optimized and the maximum yield of extraction of carotenoids attained at 2.57 ± 0.26 mg_carotenoids_ g_dried mass_^−1^ and 3.31 ± 0.02 mg_carotenoids_ g_dried mass_^−1^, for Portuguese and Brazilian algae species batch for the dried and the fresh seaweed biomass. Despite the higher extraction efficiency over the conventional (ethanol-based) method (6.48 ± 0.01 mg_carotenoids_ g_biomass_^−1^), it claimed higher selectivity towards the carotenoids (from chlorophylls).^61^

### 6.2 Pressurized system extraction

**Fig. 4:**
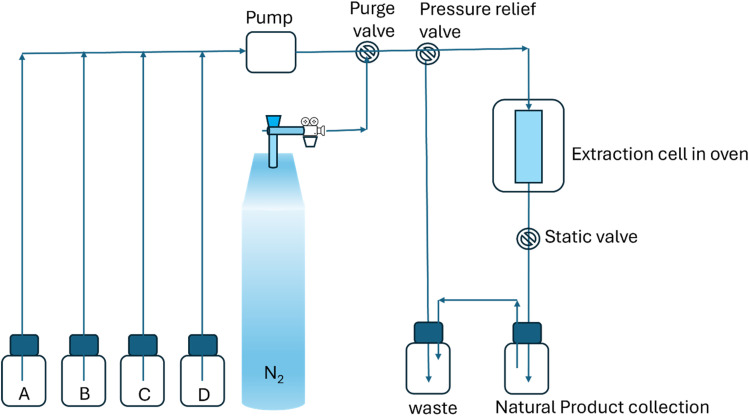
Representation of pressurized liquid extraction (PLE) technique.

A notable and emerging application of pressurized liquid extraction (PLE) is in the extraction of chemical constituents from plants and herbal materials, as depicted in Fig. 4.^99^ For example, the use of water as the solvent for PLE of Taxol or paclitaxel from yew tree bark.^100^ Additionally, PLE has been employed for the extraction of bioactive compounds such as berberine, aristolochic acids (I & II),^101^ and ginsenosides^102^ from *Coptidis rhizoma* (*huang-lien*) and the roots of American ginseng medicinal plants, respectively.

The selection of an appropriate solvent system is crucial for optimizing the PLE process.^103,104^ Recently, various research studies were performed to present the feasibility and advantages of employing aqueous non-ionic surfactant solutions as the solvent for PLE (Fig. 4).

To enhance the recovery of marker compounds from *Radix Conenoses pilosula* (*R. C. pilosula*), a method was developed that integrates surfactants such as SDS or Triton X-100 with pressurized hot water extraction (PHWE) at 95 °C.^105^ The potential of aqueous non-ionic surfactant solutions as alternative solvent systems in PLE has been previously demonstrated, using American ginseng roots as model substrates.

In 2006, a similar approach utilizing SDS and Triton X-100 in PLE was employed at room temperature and without a back pressure regulator, for the rapid determination of glycyrrhizin from *Radix glycyrrhizae* and ephedrine from *Herba Ephedrae*.^67^ In the quantitative extraction of cholesterol from solid food samples, extraction efficiency was optimized at a flow of 1.5 mL min^−1^, under an applied pressure of 10–20 bar with an extraction time of 45–50 min. Furthermore, Triton X-100 added to the extraction cell before SFE was shown to enhance the quantitative extraction of cholesterol.^106^ In the ultrasonically assisted extraction of ginsenosides from American ginseng, the use of aqueous solutions containing 10% Triton X-100 as the extraction medium resulted in faster kinetics and higher recovery rates compared to methanol or water.^107,108^

**Fig. 5:**
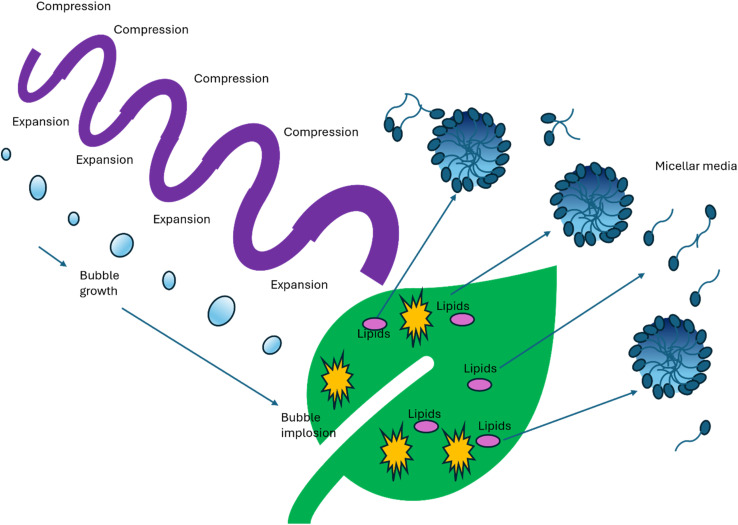
Representation of negative pressure cavitation extraction (NPCE) mechanism.

In 2020, a novel surfactant-assisted negative pressure cavitation extraction (NPCE) technique was developed (Fig. 5), utilizing the natural surfactant tea saponin to simultaneously extract seven target flavonoids from *Hibiscus manihot* L. flowers. The extraction process was optimized to establish ideal parameters: 60% ethanol containing 0.5% (w/v) tea saponin as the solvent, a negative pressure of 0.07 MPa, a liquid-to-solid ratio of 53 mL g^−1^, an extraction temperature of 61 °C, and an extraction time of 16 minutes. Under these conditions, the maximum total extraction yield was around 20 mg g^−1^.

Additionally, the tea saponin extraction solution was recyclable for at least three cycles, achieving extraction yields of 19–15 mg g^−1^ and recovery rates between 78% and 87% over repeated uses.^109^

### 6.3 Ultrasonic-assisted extraction (UAE)

The combination of surfactants with ultrasound technology enhances the penetration of solvents into plant or microbial matrices. Ultrasound waves create cavitation bubbles that, when they collapse, generate high shear forces, disrupting cell structures and releasing bioactive compounds (Fig. 2).

Bioactive compound gets into the surfactant micelle, and the efficiency of the extraction by a surfactant is increased due to ultrasonic waves (Table 4).

**Table 4:** Techno-economic summary for surfactant-assisted extraction systems

Parameter	Surfactant micellar extraction	Surfactant-UAE	Surfactant-MAE	Microemulsion systems
CAPEX	Low	Moderate	Moderate	High (formulation + separation)
OPEX	Low	Moderate	Moderate–high	Moderate
Energy requirement	Low	Low–moderate	High	Moderate
Solvent savings	High	High	Moderate	High
Environmental compliance	High (especially with biosurfactants)	High	Moderate	High
Surfactant reusability	Moderate (requires recovery system)	Moderate	Moderate	Low (formulation-specific)
Ease of integration	High	Moderate	Moderate	Low to moderate
Industrial readiness (TRL)	7–8 (near commercial)	6–7 (pilot/demonstration)	5–6 (lab-pilot)	4–6 (research-early pilot)

Studies with ultrasonic extraction using organic solvents revealed that water containing Triton X-100 at concentrations exceeding its CMC, combined with elevated temperatures, achieved comparable yields of pharmacologically active compounds from ginseng roots.^105^ The compounds like 6-gingerol, 8-gingerol, zingerone, 10-gingerol, and 6-shogaol, ultrasonic extraction coupled with surfactant micelles from gingers, were studied in 2017. Surfactants were used to solubilize solid particles in an aqueous medium and facilitated the separation of active ingredients. The results showed that the average recoveries ranged from 87.3 to 103.1% with 3.8–8.1 ng mL^−1^ limits of detection.^110^

**Fig. 6:**
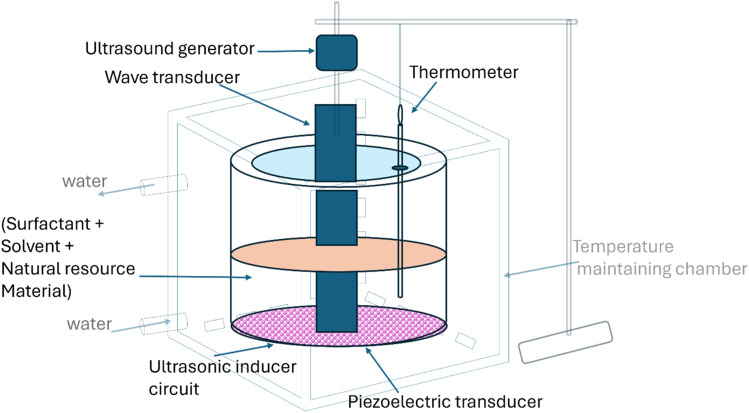
Representation of ultrasonic extraction equipment.

In 2013, a Brij/water surfactant system was employed in an ultrasound bath-assisted extraction method to isolate polyphenolic compounds from apple fruits (Fig. 6). Extraction parameters were optimized at a surfactant concentration of 7 mM, pH 3, and an ionic strength was achieved by adding a 2% (w/v) potassium chloride salt solution. This study also highlighted the analytical challenges of measuring anthocyanins in the presence of surfactants due to turbidity issues.^111^

### 6.4 Microwave-assisted extraction (MAE)

MAE uses microwave energy to heat solvents in the presence of surfactants, which increases mass transfer and improves extraction efficiency. This method is particularly effective in reducing the processing time and energy consumption associated with traditional extraction methods (Table 3).

A sustainable and integrated natural surfactant-mediated MAE technique enhances the extraction of phytochemicals from plants as depicted in Fig. 6.^112^

### 6.5 Microemulsion induced

Though surfactant-based microemulsions provide a versatile and efficient method for extracting active ingredients from natural sources, their use in the extraction of specific active ingredients from natural resources at a large industrial scale is limited. These microemulsions consist of water, oil, surfactants, and sometimes cosurfactants, creating a stable, homogeneous system that effectively solubilizes both hydrophilic and lipophilic compounds.^113^ The surfactant molecules reduce surface tension, improving the penetration of the solvent into plant tissues and facilitating the release of active compounds (Table 3). This method offers high extraction efficiency, selectivity, and stability, which is beneficial for isolating delicate bioactives like polyphenols, flavonoids, and essential oils from various plants. By optimizing surfactant concentration and system parameters, this approach can be tailored to specific extraction needs, making it valuable in the field of natural product extraction.^114,115^ The mixture of surfactants, including (saponins + Twin 80 + Span 80) and (saponins + lecithin), was studied to optimize their ability to form microemulsions.^116^

## 7. Challenges and future perspectives

Surfactants, particularly those integrated with novel technologies, present significant advantages for the extraction of bioactive compounds from natural resources due to their ability to enhance solubility and facilitate the separation of active ingredients from complex matrices. However, several challenges persist in the implementation of surfactant-based extraction processes.

One critical challenge is the selection of an appropriate surfactant. The surfactant must be compatible with both the target bioactive compounds and the matrix from which they are extracted. Its efficiency in promoting extraction must be balanced with its potential to cause denaturation or degradation of sensitive compounds, as well as the risk of introducing impurities into the final extract. Additionally, the surfactant concentration must be optimized to maximize extraction efficiency while avoiding excessive use that could lead to undesirable outcomes.

A further concern lies in the regulatory implications of surfactant use. Many synthetic surfactants, while effective, may pose toxicity risks to human health and the environment. Regulatory bodies necessitate thorough assessments of these compounds to ensure safety and sustainability. Therefore, the environmental impact and biodegradability of surfactants must be rigorously evaluated to mitigate adverse effects during both the extraction process and subsequent disposal of waste materials.

Despite the versatility and efficiency of surfactant-based extraction, scalability remains a significant hurdle. The translation of laboratory-scale methodologies to industrial-scale manufacturing is often constrained by challenges in maintaining consistency, efficiency, and cost-effectiveness when scaling up.

Future research should prioritize the development of more sustainable, “greener” surfactants. These surfactants should not only reduce environmental toxicity but also retain or improve extraction efficiency. Additionally, the integration of surfactant-assisted extraction with other advanced technologies such as SFE and nanotechnology presents a promising avenue for enhancing the extraction yields of bioactive compounds. These hybrid extraction techniques can facilitate the recovery of high-value bioactive compounds more effectively while reducing solvent usage and minimizing environmental footprint.

The combination of surfactant-based extraction with these advanced methods holds the potential for creating robust, multi-modal extraction technologies that can be applied across a broad spectrum of natural product extractions. To realize the full potential of these integrated technologies at the manufacturing scale, further research and the application of advanced engineering tools are needed to optimize process parameters and ensure seamless scale-up. This will enable the widespread adoption of efficient, eco-friendly extraction processes in industrial applications.

Surfactant-assisted extraction systems incur varying raw material costs. Common surfactants like Tween 20, SDS, CTAB, and Triton X-100 are affordable at the lab scale. However, it becomes costly at industrial volumes. Biosurfactants, though environmentally favorable, are 5–10 times more expensive due to complex production. Surfactant-assisted extraction systems reduce the use of hazardous organic solvents, significantly cutting costs related to solvent procurement and disposal.^118^

Energy use is lower in surfactant-assisted extraction methods like micellar extraction and ultrasound-assisted extraction, which often operate at ambient temperatures. These techniques can yield 30–60% energy savings compared to traditional solvent-based methods. Equipment needs vary—micellar systems require minimal investment, while the above two techniques, and continuous-flow setups, need specialized (and costlier) infrastructure. Foaming, surfactant degradation, and waste handling also add to maintenance costs.^117^

Surfactant residues must meet safety standards set by FDA, EFSA, and REACH, especially for food, cosmetic, and pharmaceutical applications. Non-ionic surfactants (*e.g.*, Tween series) are generally recognized as safe (GRAS), while anionic/cationic types like SDS and CTAB often require costly purification due to toxicity concerns.^119^

Environmental regulations favor biodegradable, low-toxicity surfactants. Biosurfactants align well with green chemistry goals but face cost and supply challenges, limiting widespread industrial adoption.

Surfactant-assisted extraction can be integrated into existing production lines for food-grade and cosmetic extractions.^120^ Micellar systems are modular and suit batch or semi-continuous setups. However, removing or recycling surfactants remains a challenge; techniques include membrane filtration,^121^ precipitation, and adsorption.^122^ Closed-loop recycling systems offer cost and compliance benefits.^123^Table 4 represents a techno-economic summary for surfactant-assisted extraction systems.

In a Southern European pilot, Tween 80 was used to extract flavonoids from citrus peel, increasing yield by 35%, reducing solvent use by 70%, and achieving a payback period under three years, though foam and residue management were issues.^124,125^ Very recently, another case involved the use of pulsed electric field treatment followed by biphasic solvent extraction to enhance lipid recovery from microalgae lipid extraction from microalgae for biodiesel.^126^ Such modifications provided ∼40% higher recovery and 50–60% energy savings. The process reached technology readiness level 6 but required improved emulsification handling.

Micellar extraction stands out as one of the most scalable and cost-effective surfactant-assisted extraction methods, making it particularly suitable for commercial applications in the food, cosmetic, and pharmaceutical industries. Techniques enhanced by ultrasound and microwave energy can further improve extraction yields; however, they require precise control and involve higher capital investment.^127^ While biosurfactants are currently more expensive than synthetic alternatives, they offer significant advantages in terms of regulatory acceptance and environmental sustainability. As production technologies advance, their economic feasibility is expected to improve. To fully evaluate the industrial potential of surfactant-assisted extraction, further techno-economic modeling and life cycle assessments are necessary, particularly to compare its performance and sustainability with alternative methods like supercritical CO_2_ and enzymatic extraction at scale.^128^

## 8. Conclusion

Surfactants are indispensable agents in the extraction of bioactive compounds from natural resources due to their ability to enhance extraction efficiency through several mechanisms. By reducing surface tension, surfactants promote increased solubility of hydrophobic compounds, thereby facilitating their release from complex matrices such as plant material, soils, or other natural sources. This action not only accelerates the extraction process but also improves the yield of active ingredients, making surfactants essential in obtaining high-quality natural products.

As the global demand for natural products continues to rise, there is a growing emphasis on the development of sustainable and environmentally friendly extraction methods. In this context, surfactants, especially those that are biodegradable, non-toxic, and derived from renewable sources, will play a pivotal role. When combined with other advanced extraction technologies, such as SFE, MAE, or ultrasonic-assisted extraction (UAE), surfactants can provide a synergistic effect, enhancing the overall extraction efficiency while minimizing the environmental impact associated with traditional solvent-based methods.

The effective integration of surfactant-based extraction techniques with other green technologies addresses several critical challenges faced by the extraction industry. This includes a reduction in the use of harmful solvents, which are often toxic and require complex disposal procedures. Moreover, the combination of these methods increases the sustainability of the extraction process by reducing the carbon footprint associated with energy-intensive traditional methods and minimizing chemical waste. Additionally, the use of sustainable surfactants can improve the selectivity of the extraction process, ensuring that only the desired bioactive compounds are extracted, thereby reducing the co-extraction of impurities that may compromise the purity of the final product.

Cost efficiency is another important advantage of surfactant-based extraction techniques. By optimizing the use of resources, such as solvents and energy, these methods can lead to lower operational costs in comparison to traditional methods. Furthermore, surfactant-assisted extraction processes can enhance the recyclability and recovery of raw materials, allowing for more efficient use of natural resources and reducing waste generation.

One of the major challenges for industrial adoption of these advanced extraction methods is the scale-up from laboratory to manufacturing levels. To successfully transition from small-scale experimental systems to large-scale production, advanced engineering expertise is required to optimize process parameters, ensure uniformity, and maintain the efficiency of the combined extraction techniques at a larger scale. This will involve addressing issues such as mass transfer, equipment design, and process control to ensure that the benefits observed at the laboratory scale are consistently realized in industrial applications.

The successful engineering and scale-up of efficient, surfactant-based extraction combinations will revolutionize the natural products industry. By providing more sustainable, cost-effective, and environmentally responsible methods for extracting bioactive compounds, these innovations have the potential to meet the growing global demand for natural products while contributing to the development of a greener, more sustainable extraction industry.

## Conflicts of interest

There are no conflicts to declare.

## Data Availability

This is a review article and no new data were generated or analysed in this review article. Hence, data sharing is not applicable to this article.
